# From Lizard to Snake; Behind the Evolution of an Extreme Body Plan

**DOI:** 10.2174/138920212800793302

**Published:** 2012-06

**Authors:** Joost M Woltering

**Affiliations:** University of Geneva, Department of Genetics and Evolution, 30 quai Ernest Ansermet, 1211 CH, Genève, Switzerland

**Keywords:** Snake, caecilian, body plan, hox, somitogenesis, limb, evo-devo, body elongation.

## Abstract

The elongated, snake-like skeleton, as it has convergently evolved in numerous reptilian and amphibian lineages, is from a developmental biologist’s point of view amongst the most fascinating anatomical peculiarities in the animal kingdom. This type of body plan is characterized by a greatly increased number of vertebrae, a reduction of skeletal regionalization along the primary body axis and loss of the limbs. Recent studies conducted on both mouse and snakes now hint at how changes inside the gene regulatory circuitries of the *Hox* genes and the somitogenesis clock likely underlie these striking departures from standard tetrapod morphology, suggesting scenarios by which snakes and other elongated species may have evolved from more ordinarily bodied ancestors.

## FROM *GENESIS* TO ONTOGENESIS; THE CASE OF THE SNAKE

‘*Then the Lord God brought the three of them to judgment, and he said to the serpent ‘Because you have done this, cursed are you above all cattle and above the beasts of the field. Upon your belly you shall go about, and your feet shall be cut off, and you will cast of your skin once every seven years, and the poison of death will be in your mouth, and you shall eat dust all the days of your life*.’(Genesis 3.14, Targum PS. Jonathan [1]). In addition to a description of the world’s creation and eviction from paradise, the biblical genesis epos *en passant* also provides, with the passage cited above, an explanation for how the snake received it’s striking features as a punishment for talking Eve into eating from the apple, thereby most likely giving the earliest known account of an ‘evo-devo conundrum’; that is, *avant la lettre* and, as will go without saying, not quite up to modern scientific standards. Nevertheless, as this goes to show, mankind appears to always have had a certain fascination with snakes, probably related to the threat their poison poses, something which even has left an evolutionary imprint on our neuronal wiring in the form of a strong predisposition to fear of snakes [2]. In the last several years a number of important studies have appeared, conducted on both model organisms and on snakes, that do offer a scientific explanation for the development and evolution of what everyone immediately will recognize as a *serpentiform* body plan [3-6]. As frequently in the field of evo-devo, insight into the developmental basis of peculiarities in ‘non-model organisms`, lacking advanced experimental protocols and with typically limited opportunities for acquisition of experimental material, relies heavily on the progress made in unraveling basal developmental mechanisms in model species such as mice and chicken (see for instance [7,8]). These mechanisms then in turn lend themselves for a comparative approach in species of interest.

Recently such studies have probed the gene regulatory network of the somitogenesis clock [3] and the *Hox* genes in snakes [4,5] and, concerning the *Hox *genes, to a lesser extent in caecilians [4], an amphibian order that convergently evolved a snake-like appearance. Meanwhile studies in the mouse have provided important new insights into how the *Hox* genes mediate the formation of ribs [9] and play an essential role in the termination of body axis extension [6], two processes which are obvious candidates for deviations in snakes. What follows is a discussion of how departures in these developmental mechanisms likely underlie the formation of a *serpentiform *body plan. With this information it is now possible to assess how these processes contribute to various types of body elongation differently and to reconstruct the scenarios by which snakes may have evolved from lizard-like ancestors, while taking developmental, anatomical and paleontological data into consideration. 

## THE* COULEUR LOCALE* OF THE VERTEBRATE SKELETON

**Fig. (1). Comparison of mouse and snake body plans.**
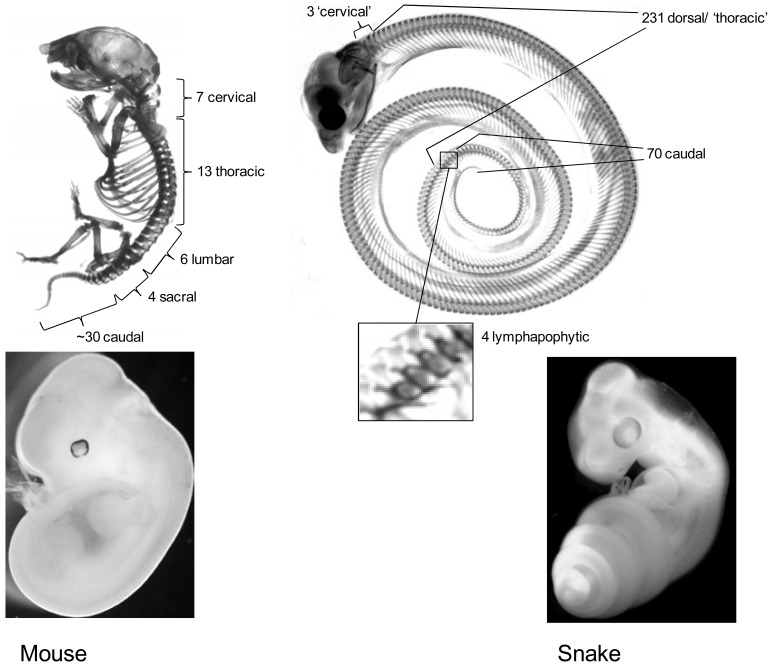
Upper panel: skeletons of a newborn mouse and a hatching stage corn snake. The mouse provides an appropriate example of the regionalization that has evolved in the tetrapod axial skeleton, which consists of various different specialized vertebrae types and possesses limbs positioned at fixed positions along the antero-posterior axis. In comparison, the snake has a huge increase in vertebrae number, lacks limbs, displays an extensive homogenous rib-bearing region (dorsal vertebrae/thorax) and lacks a rib-less lumbar region. Close up is shown of the 4 lymphapophyses bearing vertebrae. These ‘forked ribs’ form a snake specific adaptation and serve to protect lymphatic hearts. Lower panel: a day 12 post fertilization mouse embryo and a ~ 2 days post ovo-positioning corn snake embryo are shown.

The vertebrate axial skeleton is strongly regionalized along its anterior posterior body axis [10-12] (see Fig. **1**). Typically, different axial positions have their own specialized type of vertebrae resulting in a functionally coherent skeleton that combines adaptations for protection, stability and agility, overcoming their at times conflicting anatomical requirements. These specializations have most strongly evolved in the mammalian skeleton which is subdivided into distinct cervical (neck), thoracic (thorax, rib bearing), lumbar (posterior to the thorax, lacking ribs), sacral (connecting the vertebral column to the pelvis) and caudal (tail) regions [10-12]. These different domains clearly demonstrate how specialized vertebrae function at their own position along the axis. For instance: the atlas-axis complex in the cervical region allows for a flexible attachment of the head onto the spinal column while the ribs in the thorax protect vital organs and supply an attachment for the diaphragm without which breathing would be problematic. In turn their absence in the lumbar and cervical regions prevents unnecessary interference with body movements. Such adaptive regionalization is to some extent present in all vertebrates and numerous species have evolved lineage specific adaptations. In this context it is worth mentioning the vertebrae that contribute to the turtle’s carapace [13], the fusion of caudal vertebrae into the urostyle in frogs which evolved as an adaptation to jumping [14], the hugely elongated ribs supporting lateral skin flaps used for flight of the lizards from the genus *Draco *[15] and the sacral lymphapophyses (‘forked ribs’) of snakes which enclose the lymphatic hearts [16].

When referring to reptiles or amphibians, it is important to bear in mind that, strictly spoken, it is incorrect to use the mammalian nomenclature for thoracic and lumbar regions [16]. Instead, the pre-sacral reptilian skeleton is divided into cervical and dorsal regions [16]. The cervical region is located anterior to the shoulder girdle and can consist of both rib-less as well as rib-bearing vertebrae whose ribs are never connected to the sternum. The dorsal region extends backwards from the first vertebra bearing a rib that is fused with the sternum to the sacral region, including any pre-sacral rib-less vertebrae. Therefore reptiles do not possess true thoracic or lumbar vertebrae but rib-less or rib-bearing dorsal vertebrae and the presence of ribs on anterior vertebrae does not exclude them from being cervical. However, considering the expected similarities in underlying developmental patterning mechanisms, which have predominantly been characterized in mammals, I will, for sake of clarity, adhere to the mammalian terminology, referring to all rib-bearing vertebrae as thoracic and all pre-sacral rib-less vertebrae as lumbar.

## THE BODY OF THE SNAKE, WHAT’S NEW?

As an adaptation to a burrowing and crawling lifestyle the transformation into an elongated ‘snake-like’ body plan has occurred repeatedly during vertebrate evolution [10-17]. In extant *Tetrapods* (land animals) there are numerous lineages in which this transformation has occurred independently and therefore it is one of the most striking recurrent modifications in the vertebrate body plan. In reptiles and amphibians this elongated body type evolved convergently in snakes,* amphisbaenians*, *anguidae*, lialis and *acontias*, caecilians, *amphiuma *and *siren* species. The transition from a standard to a long body shape is accompanied by a huge increase in vertebra count; snakes have >300 vertebrae compared to ~65 for lizard (*Anolis carolinensis*) and 60 for mouse [18]. Effectively the addition of vertebrae thus provides the basis for the elongation in snakes. It is however not only elongation that marks these species; the transition into a snake-like body form is characterized by further dramatic changes in the morphology of the axial skeleton, including homogenization and extension of the thorax at the expense of a lumbar region and the disappearance or reduction of limb, shoulder girdle and pelvic structures. Because of its uniformity, the lack of landmark structures such as limbs and pelvis and the absence of clear vertebral specialization in the pre-caudal region, this type of body plan is often described as being ‘deregionalized’. The snake-like body plan is thus characterized by two landmark changes; an extension of the primary body axis by the addition of more vertebrae and a deregionalization of the skeleton involving a reduction of the vertebral specialization along the antero-posterior axis (Fig. **1**). 

## DEVELOPMENT AND PATTERNING OF THE AXIAL SKELETON

During embryogenesis most of the mesoderm becomes subdivided into discrete metameric segments, the somites, through the process of somitogenesis which takes place in close coordination with the posterior growth of the embryo by cell proliferation within the tail bud [19]. The somites give rise to most of the axial skeleton and associated muscular apparatus. Except for the first 5 somites, which will contribute to the skull, the somites give rise to vertebrae and the number of somites formed during embryogenesis therefore determines the number of vertebrae in an organism (save exceptions such as frogs that resorb most of the caudal somites upon metamorphosis [20]). The somites are repetitive units sharing a similar process of ontogenesis and with an initially similar appearance, yet developing into distinctly different types of vertebrae depending on their positions in the skeleton. This regionalization is established through differential expression of the *Hox* genes along the antero-posterior axis [19,21-26].

In tetrapods the *Hox* genes are organized in 13 paralogous groups organized in four clusters (*HoxA*-*HoxD*) resulting from the two rounds of genome duplication early in vertebrate evolution. Not every paralogous group is represented in each cluster (though minimally twice in total) due to frequent gene loss during evolution. Typically genes from the same paralogy group are expressed in similar domains and act redundantly in specifying axial identity, something which severely confounds mutant analysis which usually has to resort to full paralogy knockouts (see for instance [27, 28]). 

The *Hox* genes are expressed collinearly along the axis in line with their position in a *Hox* cluster; genes located at one end of a cluster and carrying a low number (e.g. *HoxA1*) are expressed more anteriorly than genes located at the opposite end of a cluster which carry a high number (e.g. *HoxA13*) [22,29,30]. During embryogenesis transcription of the genes becomes activated in the gastrula and later the tail bud in a temporal collinear sequence during which anterior genes are activated earlier than posterior genes [31-33]. 

Typically anterior *Hox *genes extend their expression posteriorly along the axis into regions where their activity appears to be overruled by the action of more posterior *Hox* genes according to the principle of ‘posterior-prevalence’ which predicts that posterior genes are phenotypically dominant over anterior genes when both are expressed together [34]. The phrase ‘*Hox *code’ has been coined to refer to the combination of *Hox* genes expressed at a particular axial level [35]. The expression of *Hox* genes has been shown to correlate with the same anatomical transitions in different species [36,37] and the regulation of these genes has therefore been considered a key candidate mechanism for evolutionary modifications of the body plan.

## ELONGATION, WHERE A FASTER TICKER MAKES MORE SOMITES

**Fig. (2). Increased frequency of the somitogenesis clock oscillator results in an increased somite number in snakes.**
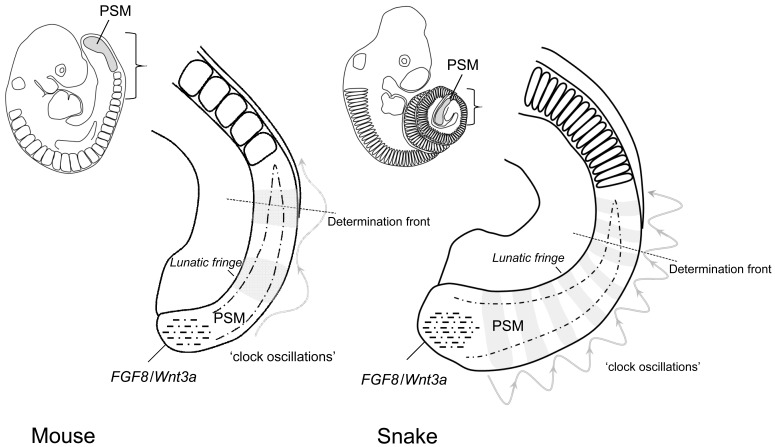
Somites are formed in the pre-somitic mesoderm (PSM) in the tailbud through interplay between the oscillating genes of the somitogenesis clock (indicated by a wave function and the banded expression of *Lunatic fringe* in grey) and an *FGF8/Wnt3a* gradient extending anteriorly from the tailbud (dash-dotted lines and triangle). At a certain position in this morphogen gradient, cells become competent to respond to genes of the somitogenesis clock to form prospective somite boundaries, which will bud off slightly later when cells have moved to more anterior positions. As the oscillation frequency of the somitogenesis clock is about 4 times higher in the snake than in the mouse the determination front is ‘hit’ more frequently and more often per number of cells by expression of somitogenesis clock genes. This results in the production of more but smaller sized somites in the snake. This acceleration of the somitogenesis clock is presumably responsible for most of the increase in the snake’s vertebral number [3,38].

The first hallmark of the snake body plan is the axial extension. Snakes have a greatly increased number of vertebrae, which as stated above derive from the somitic mesoderm and snakes form a correspondingly high number of somites [18,3]. Recently, part of the mechanism behind this large somite count was elucidated in snake embryos [3,38]. During embryogenesis somites are formed at the posterior end of the embryo from the pre-somitic mesoderm (PSM) by a genetic network which operates through a ‘clock and wavefront’ mechanism [19]. This involves an elaborate interplay between a *Wnt3a*/*Fgf-8* gradient (the wave front) and the oscillating expression of various genes of the ‘somitogenesis clock’ (amongst others *Notch* and *FGF* pathway genes) (see Fig. **2**). The PSM is located at the posterior end of the body, in between the newly formed somites and the growth zone of the tail bud. At regular intervals somites bud off from the anterior end of the PSM while at its posterior end proliferation of the tail bud replenishes the population precursor cells within the PSM. Cells in the posterior PSM are maintained in an undifferentiated state by *FGF-8* and *Wnt3a *signaling derived from the expression in the tail bud and subsequently inherited as they migrate into the PSM. As these cells spend more time outside of the tail bud their relative position within the PSM shifts upwards due to the production of new PSM cells posteriorly and the reduction of the PSM anteriorly, caused by the formation of new somites. Meanwhile the *FGF* and *Wnt* signals inherited from the expression in the tail bud decay. Together these processes effectively result in the formation of a *FGF*/*Wnt* morphogen gradient along the PSM. A certain threshold concentration within the *Wnt*/*FGF *gradient now establishes the ‘determination front’ which is the position within the PSM where cells become competent to form a somitic boundary in response to signals from the somitogenesis clock. The somitogenesis clock comprises genes from the *Notch*, *Wnt* and *FGF *pathways, which are expressed in a rhythmic fashion in the PSM. Because cells are out of phase depending on their time of creation in the tail bud, the expression of the clock genes appears to occur as a wave, sweeping through the PSM, inducing the formation of a somite each time a ‘wave’ passes the determination front. The rhythm of this oscillator thus determines the periodicity with which somites are produced.

The expression of the main genes involved in somitogenesis was probed in corn snake in order to investigate the basis of its increased somite number [3]. Somites appear to form in snakes according to a conserved mechanism; the genes positioning the determination front, *FGF-8*/*Wnt3a*, are expressed comparably to other vertebrates and the expression of *MSNGN1*, whose anterior boundary marks the determination front, indicates similar dynamics of the gradient. A difference was found for *Lunatic fringe *an oscillating gene which is part of the ‘somitogenesis clock’. Its expression appears in a similar striped pattern in the pre-somitic mesoderm, each stripe representing the ‘frozen’ active phase of a ‘wave’. In snake the number of concurrent waves present in the PSM is much higher than in chicken or mice. When corrected for differences in overall developmental rate it turns out that the snake ‘clock’ ticks about 4 times faster than in mice or lizards. It thus appears that the somitogenesis clock has a higher periodicity in snakes and therefore hits the determination front more often per number of PSM cells, thereby creating more somites from the same number of precursor cells. This is consistent with the fact that snakes produce more but smaller sized somites per time unit than lizards, mice or chicken.

## AXIAL TERMINATION, WILL THE LAST *HOX *PLEASE TURN OFF THE CLOCK?

As long as the rate of somite formation and tail bud extension are in equilibrium, somitogenesis could hypothetically continue infinitely [38]. However, near the end of somitogenesis, the PSM becomes exhausted when tail bud growth slows down and ceases to produce new somite precursors (the shrinking of the PSM). At a constant somitogenesis rate, an extension of the body axis also would occur in case the shrinkage of the PSM were delayed, leading to an increase in the total number of cells produced by the tail bud. In this scenario the axis would simply continue growing for a longer time and consequently produce more segments. This was investigated in the corn snake by estimating the number of times the population of cells in the PSM is renewed [3]. There is evidence for on average 21.5 generations of PSM cells in the snake compared to only 16.3 in the mouse. Since the snake PSM uses ~30% more cells, this increase would account for only 21 of the 250 somite difference between mouse and corn snake, that is, without an acceleration of the clock relative to the mouse (i.e. 30% of the 65 mouse somite count). However, in combination with the higher speed of the clock it allows the snake an estimated gain of 77 somites (i.e. 315-((315/21.5) x 16.3)), which is about the size of the entire tail region, demonstrating that both processes are relevant for the increase in body length. Furthermore both mechanisms could have proportionally different impacts on the elongation in different species (see below).

**Fig. (3). Hox13 genes terminate axial extension and are expressed at a lower dose in the snake’s tail bud.**
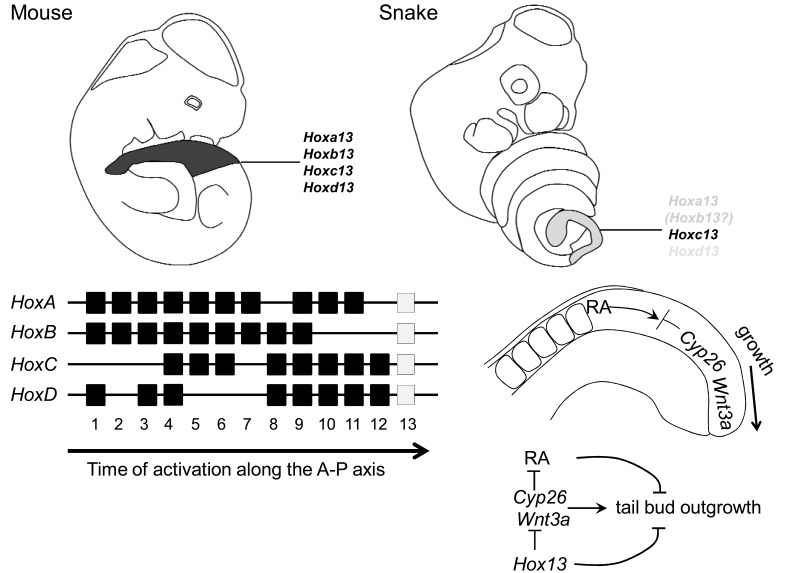
The *Hox13* paralogous genes are the final *Hox* genes to become activated in the tail bud in the temporal collinear sequence of *Hox* gene activation that occurs during development (mouse *Hox* clusters shown). In the outgrowing tail bud *Wnt3a* provides a growth stimulating signal while *Cyp26* counteracts a growth suppressing Retinoic acid (RA) signal emanating from the somites. *Hox13* genes have been suggested to terminate tail bud outgrowth by suppressing *Wnt3a* and *Cyp26* activity [6]. In the mouse four paralogous *Hox13* genes are expressed strongly in the tail bud (approximate cumulative expression domain indicated). In the snake only *Hoxc13* has comparable expression whereas *Hoxa13* and *Hoxd13* are only weakly and transiently expressed thereby providing a mechanism for prolonged tailbud outgrowth in the snake [5]. *Hoxb13* has not been characterized in the snake but since this gene exists as a pseudogene in the lizard (*Anolis carolinensis*) (JMW unpublished observations) it is possible that a functional *Hoxb13* ortholog also has disappeared from the snake genome.

A candidate signal to induce the shrinking of the PSM is retinoic acid (RA), which is produced in the mature somites by* RALDH2 *and creates a gradient opposing the *FGF*/*Wnt *signal from the tail bud. RA has been shown to counteract the growth promoting signals expressed in the tail bud and to induce apopotosis. In the tail bud the somite derived RA signal is initially buffered by expression of the *CYP26* which degrades RA and the expression of which is essential for posterior axial extension [39]. This growth arresting mechanism has now been linked to expression of the *Hox13 *group genes (Fig. **3**). These genes are the final paralogs to become transcribed in the temporal collinear sequence of activation during axial extension [29,32] and consequently their expression along the axis is restricted to the tail region. Knockout of *Hoxb13* in the mouse leads to a slight increase in body axis length [40] indicating a braking effect on body axis extension. Recently experiments precociously expressing *Hoxa13*, *Hoxb13 *or *Hoxc13 *in the PSM have further confirmed this function [6]. In these experiments, mice overexpressing *Hox13* genes produced posterior truncations in the post-sacral region and, at embryonic stages, show decreased expression of *Wnt3a* and *Cyp26a1* expression in the tail bud. It thus appears that *Hox13 *paralog genes are actively involved in the termination of posterior axial growth, possibly by creating a permissive environment for the retinoic acid signal emanating from the somites. In this manner the temporal collinear activation of *Hox13 *paralogs as the final *Hox* genes in a collinear sequence provides an elegant mechanism for timed cessation of axial elongation.

It has now appeared that alterations in this mechanism are likely involved in the delayed shrinking of the PSM and the increased generation number of PSM cells in snakes. The expression of the posterior *Hox* genes in snakes deviates from what has so far been observed in amniotes. Although the expression of *Hoxc13* appears to be normal in the tail region of the snake [4,5], the expression of two other *Hox13 *paralogs shows important differences. Although *Hoxa13* and *Hoxd13* are expressed in the developing genitals, their expression is virtually absent from the tail region (whereas their expression in lizard is readily detectable) [5]. This probably means that the snake PSM is subjected to a lower dose of *Hox13 *paralogue gene expression towards the end of somitogenesis, something which otherwise would act to terminate axial growth and thereby somitogenesis. A failure to activate a sufficiently high dose of *Hox13* paralogs in the tail bud thus probably results in the longer lifetime of the PSM as observed in snakes. In this sense it appears that in snakes the braking mechanism required to terminate posterior growth is faulty and thereby contributes to the elongation of the body axis. The mechanism behind the disturbed regulation of *Hoxa13* and *Hoxd13 *has not yet been clarified and also the status and expression of *Hoxb13*, the fourth tetrapod member of the *Hox13 *paralogous group, are unclear in the snake. Interestingly, the accumulation of transposable elements within the *squamate Hox* clusters [41,5], absent from their mammalian counterparts, may have caused the disruption of regulatory modules.

## DEREGIONALIZATION, AN ALTERNATIVE INTERPRETATION OF *HOX* CODES.

The second hallmark of the snake body plan is the skeletal deregionalization, which has evolved along the primary body axis. The vertebral specialization as found in the skeleton of most other tetrapods is largely absent from the snake’s trunk skeleton; most of the snake’s pre-caudal vertebrae bear ribs (about 230 out of 315 in the corn snake [3,4]) with negligible differences between the rib-bearing vertebrae located at different positions; a rib-less lumbar region is absent; and in addition, snakes have lost the limbs which normally develop at fixed positions along the body axis.

As stated previously, the vertebrate axial skeleton is composed of repetitive segments, namely the vertebrae, which derive from somites with a near identical appearance, though which attain highly different morphologies during development due to the various *Hox* codes expressed along the axis. The *Hox* codes responsible for the patterning of the mouse skeleton are well understood [25,42], mainly due to the single and paralogous compound mutants, which now have been reported for all *Hox* genes expressed in the pre-caudal region [43,28,44], and two studies overexpressing *Hox* genes within the PSM [45,9]. In the mouse it appears that the thoracic vertebrae are patterned by the group *Hox5 *to *Hox9* genes [44,43]. The absence of ribs in the lumbar region develops due to the suppression of rib formation by *Hox10 *genes [28,45]. Mice mutant for all 3 *Hox10 *genes develop with ectopic ribs in the lumbar region [28] while mice in which *Hoxa10* is precociously activated in the pre-somitic mesoderm lose the thoracic domain and all pre-caudal vertebrae develop without ribs [45]. 

The *Hox* code responsible for the induction of the ribs themselves is still somehow mysterious [25]. The rib inducing capacity of *Hox6* group genes has been demonstrated by overexpression assays in the PSM, which results in mice bearing ribs on all vertebrae whereas no other paralogous group tested appeared to have this capacity [9]. However, quadruple *Hox6* mutant mice do develop ribs [43] and no other *Hox* mutant combination has been reported to lead to an absence of ribs. It therefore seems plausible that* in vivo*, ribs are redundantly induced by a combination of *Hox6* paralogs together with another, yet unidentified paralogous group [25]. 

**Fig. (4). Loss of Hox10 rib-suppressing activity in the trunk of the snake.**
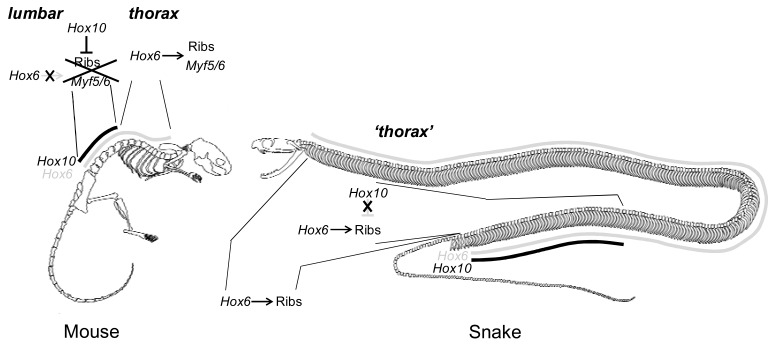
In the mouse rib-bearing thoracic and rib-less lumbar domains are determined by expression of *Hox6* and *Hox10* genes (for the apparently redundant role of *Hox6* genes in rib induction see main text). Expression of *Hox6* genes in the PSM leads to activation of *Myf5* and *Myf6* in the hypaxial domain of the somites resulting in rib formation. Expression of *Hox10* genes at the time the lumbar somites are formed disrupts this cascade and represses *Myf5* and *Myf6* resulting in rib-less vertebrae [9]. In snakes *Hoxa10* and *Hoxc10* are strongly expressed in the posterior thorax, nevertheless this region develops ribs wich are identical to ribs located more anteriorly, outside of the *Hox10* expression domain [4,5].

Pioneering investigations of *Hox* gene expression in Python embryos contributed to the hypothesis that snakes may have evolved this deregionalization by a corresponding homogenization of *Hox *gene expression domains along the axis [46] establishing the same *Hox* code in most of the pre-caudal somites which consequently would adopt identical vertebral identities. Recently the expression of ‘anterior’ and ‘posterior’ *Hox* genes has been characterized more extensively in the trunk of the corn snake [4,5]. At the anterior end of the skeleton the expression of *Hoxa3* and *Hoxb4* corresponds to the atlas and axis respectively, consistent with their expression and function in mouse while *Hoxa6 *expression coincides with the first rib-bearing vertebra [4]. The boundaries of genes with a more posterior expression within the thoracic region do however not obviously correspond to anatomical transitions in vertebral morphology (*Hoxc5*, *Hoxc6*, *Hoxa7*, *Hoxb7*, *Hoxb8*, *Hoxb9*) [4], although in the mouse these genes serve to pattern the rib cage [44,43]. Apparently a form of cryptic regionalization by the *Hox* genes exists, which however is not translated into anatomical differences in the skeleton. This situation is even more striking in the case of the *Hox10* genes. As mentioned above the expression of *Hox10 *genes is linked to the suppression of rib formation, as is now understood through suppression of *Myf5* and *Myf6* in the hypaxial part of the somite [9]. The snake lacks a lumbar, rib-less pre-caudal domain and would thus be expected not to express *Hox10 *genes within this region. However, strong expression is observed for *Hoxa10* and *Hoxc10* within the thoracic region [4,5], which seems inconsistent with the rib-suppression role of these genes in the mouse. It thus appears that the somites that will give rise to the homogeneous rib-bearing vertebrae of the snake thorax are regionalized by expression of *Hox* genes whereas this is not translated into regionalization of the skeleton (Fig. **4**). Together, these observations suggest that the deregionalization within the snake pre-caudal region results from an inability to conventionally interpret the *Hox* codes within the somites rather than from a homogenization of *Hox* expression domains as originally proposed [46]. It was observed that several of the trunk *Hox *genes have atypical gradual anterior limits of expression (e.g. *Hoxc6* and *Hoxb9*), which indeed may indicate that a disconnection from vertebral identity has lead to a relaxation of their expression with a sharp transitional boundary between somites [4].

This alternative interpretation of axial *Hox* codes could have evolved at any of several levels; the most probable candidate mechanisms are the evolution of *cis*-regulatory elements in target gene promoters [4] and changes in protein coding sequences [4,5], comparable to the changes in the *Ubx *protein coding sequence which are responsible for evolution of the invertebrate body plan [47,48]. Indeed it appears that the coding sequences of *Hoxa10* and *Hoxc10* show an accelerated evolution in snakes [5]. It now awaits testing in mouse models to which extent these genes have preserved their rib suppressing capacity. The identification of *Myf5* and *Myf6* as downstream direct targets of *Hox6 *and *Hox10* group genes [9] now opens interesting avenues to investigate the role of changes in *cis*-regulatory sequence evolution in the alternative interpretation of the *Hox* codes inducing rib formation and rib suppression.

## AS IF TORN LIMB FROM LIMB

Another important feature of the *serpentine* body plan is the absence of limbs. Limb loss or reduction is a frequent phenomenon during reptile evolution that is strongly linked to body elongation. In lizards the transition from normally limbed species into limbless taxa is well documented and has been shown to occur in as little as a few million years. Primitive snakes such as Boas and Pythons do possess vestigial hindlimbs at the position of the cloaca [46], however no indications have ever been discovered for forelimb specification in snakes. 

Limbs develop in a competence region of the lateral plate mesoderm (LPM) in response to a signal from the somites, possibly retinoic acid [49]. The *Hox* genes are popularly believed to be involved in the positioning of the limbs along the axis at the position of the shoulder girdle and at the position of *Hox9* anterior boundaries in the LPM. The loss of limbs in snakes has originally been attributed to the homogenization of the *Hox* codes along the axis resulting in an overpatterning of the *Hox* code determining their original position [46]. First, it needs to be remarked that the existence of such a *Hox *code for limb positioning is doubtful. A correlation between the expression of combinations of *Hox* genes and the positioning of the limbs exists in somitic mesoderm and LPM but if and how *Hox* genes are actively involved in determining the axial positions for limb bud formation remains extremely elusive. *Hox9* genes were considered a candidate signal determining the positioning of limb buds in the lateral plate [50], it has now been shown that combined loss of function, although surprisingly affecting forelimb autopod development (see below), does not affect their position along the axis [27]. In addition, in lizards, the sequence to limb reduction evolves *via *consecutively smaller limbs, which however develop at approximately stable positions at the neck-trunk transition and around the cloaca. Even in completely limbless lizards such as the Slowworm (*Anguis fragilis*), remnant limb buds still develop at the expected anterior and posterior positions [51]. The only evidence possibly indicating a limb reduction mechanism operating through alteration of positional information along the axis either *via Hox* codes or *via *a downstream interpretation was reported for the corn snake [4]. First, it was observed that, whereas the somitic mesoderm and the LPM in mouse typically have different *Hox* codes with the anterior boundaries for the *Hox* expression being offset (see for instance reference [43]), in the corn snake they have coinciding anterior boundaries. Second, the forelimb marker *Tbx5*, which normally in mouse becomes restricted to a forelimb domain during development, is expressed in an atypical pattern throughout the LPM of the trunk in the corn snake despite a regionalization of the LPM by *Hox* expression domains. However, because the *Hox* codes in somitic mesoderm and LPM and the upstream signals governing *Tbx5* expression and their possible connection to *Hox *expression are not well understood it is very difficult to interpret these results. 

Nevertheless, there is a strong correlation between limb loss and body elongation [17], suggestive of a developmental constraint on the ontogeny of limbs in elongated taxa. The developmental mechanism behind this limb reduction however remains elusive. Most evidence indicates that limbs remain specified at the appropriate antero-posterior position during the evolutionary sequence into fully limbless forms. In limb reduced species this appears due to a degeneration of the limb bud during development. An intriguing observation is that distal forelimb identity becomes established though the expression of *Hox9* genes in the flank [27] through the combined activity of all *Hox9* genes and alterations in the combined boundaries of *Hox9* genes in the LPM could be potentially related to the frequent observed digit reductions associated with body elongation. 

## A BODY OF VARIATION AND CONVERGENCE

Developmental data now implicate changes within three different mechanisms in the evolution of a snake-like body plan. These are: an accelerated pace of the somitogenesis clock [3,38], a delayed shrinkage of the PSM by inadequate activation of *Hox13* genes [5] and an alternative interpretation of *Hox *codes in the pre-caudal region [4,5]. As mentioned earlier, the elongated body plan is a striking example of evolutionary convergence, which has evolved independently in numerous species. To what extent do the mechanisms discussed here underlie this anatomical convergence? In most of these species, solving this issue will be hampered by the possibilities to obtain sufficient embryonic material to extensively probe these genetic pathways. However, for some points data is already available and for others educated guesses can be made based on circumstantial evidence. 

For instance, the accelerated pace of the somitogenesis clock in snakes results in the formation of more but also smaller sized somites [38]. Although it will be challenging to obtain sufficient material to investigate all the components of the somitogenesis pathway in other elongated species, comparison of somite size may give a good indication of their relative clock speeds [38]. Embryos of the slow worm (*Anguis fragilis*) compared to the green lizard (*Lacerta viridis*) do indeed develop more and smaller somites indicating a corresponding frequency increase in the somitogenesis clock (Raynaud, 1994 from reference [38]). 

In caecilian embryos the expression of a number of *Hox* genes has been investigated [4]. Pre-caudal genes appear to be expressed in a manner very similar to their snake homologs resulting in a cryptic regionalization of the trunk, which is not translated in obvious differences in thoracic vertebrae. Most interestingly *Hoxc10 *is expressed with an anterior boundary well within the thorax. It thus appears that a similar insensitivity to the *Hox *codes has evolved in caecilians as in snakes [4].

**Fig. (5). Disproportional elongation of trunk and tail regions occurs in different taxa.**
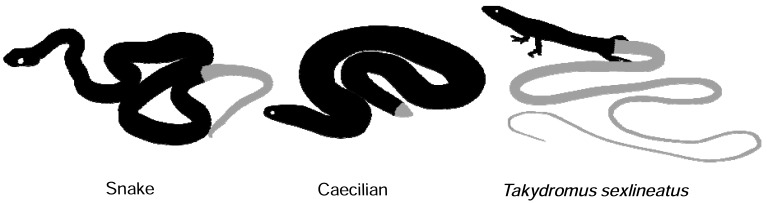
Whereas snakes display elongation in trunk and tail regions (see also main text on the different snake ecotypes), caecilians have an elongated trunk while possessing an extremely short tail comprising several vertebrae only. Another striking example of disproportional body elongation is the lizard *Takydromus sexlineatus,* which has evolved a tail many times the length of the trunk, which has remained essentially normal. The behavior of trunk and tail elongation as independent evolutionary modules suggests different developmental mechanisms behind these two processes.

Striking differences exist with respect to the axial elongation between snakes and caecilians; in snakes the axis continues its extension after *Hox13 *transcriptional activation at the trunk tail transition. Beyond this point another ~75 vertebrae are formed which all express *Hoxc13 *[4,5]. Caecilians in contrast form only 5 caudal vertebrae while *Hoxc13* is also activated at the trunk tail transition [4]. This may indicate an efficient termination of axial growth as soon as *Hox13* genes become expressed; it seems plausible that an extended lifetime of the PSM does not contribute to the elongation of the caecilian body plan. Indeed, it has been proposed that trunk and tail elongation are dissociated characters (Fig. **5**); snakes have evolved fossorial, terrestrial and arboreal ecotypes which differ in trunk-tail ratios [52]. Similar observations have been made with respect to body elongation in lizards where two ecomorphs can be distinguished: short-tailed burrowers or long-tailed surface dwellers [53]. The uncoupling between trunk and tail length is even more obvious in species with an increased length of their tail only, such as the lizard *Takydromus sexlineatus* (>100 tail vertebrae) that retains a normal trunk. The behavior of these characters as separate evolutionary modules can now be understood by the two different mechanisms involved. An acceleration of the somitogenesis clock is expected to result in an increase in somite number, which would be reflected in all body regions equally. (Although there may be a bias towards trunk elongation due to the slowing down of the clock towards the end of somitogenesis [3], still this would affect the proportionality in different similarly elongated species to the same extent). However, an increased lifetime of the PSM by insufficient activation of *Hox13* genes at the trunk-tail transition will affect the length of the tail only. These processes together provide a highly flexible scaling mechanism for the body plan. In caecilians, which do have an extremely high trunk-tail ratio, it even seems plausible that the rate of PSM shrinkage has *increased* as compensation (possibly by higher *Hox13* gene levels of expression) in order to develop a short tail despite a higher speed of the somitogenesis clock.

## DID SNAKES STICK OUT THEIR NECK?

Inescapably, snakes must have evolved from reptiles with a ‘standard’ body plan. On this point the fossil record is however highly fragmented and therefore the sequence of morphological transitions which took place during snake evolution is elusive [54,17]. The oldest known snakes stemming from the cretaceous [55,56] appear decisively snake like in appearance with few if any hints to the ancestral organization of the pre-cloacal region. In addition no undisputed transitional forms between lizard-like animals and snakes have ever been uncovered. Regarding the evolution of snakes two scenarios have gained popularity; a terrestrial origin through the evolution from fossorial lizards [57,58] or an aquatic origin [59,56, 60-63]. Indeed both environments are expected to favor the evolution of body elongation *via *the accompanying locomotory advantages. In extinct aquatic lizards body elongation through cervical and dorsal elongation has been observed in *Dolichosaurus* and* Adriosaurus*, aquatic lizard species with long necks and estimated cervical counts between 10 and 19 [17, 59, 60] and a similar trend involving cervical elongation has been speculated to have occurred in snake ancestors living in an aquatic environment. Evolution of body elongation in a fossorial environment has been well documented [64, 53] and does not expectedly favor transitional forms with an elongated neck region. In this context the length of the snake’s neck is of interest. Here it is important to remember that, in contrast to mammals, reptilian cervical vertebrae are frequently rib-bearing (see also the previous discussion on the incorrect use of the mammalian nomenclature in reptiles), hence the presence of ribs is not a good indicator of the division between neck and trunk regions, which is largely made on basis of the positioning of the shoulder girdle, sternum and forelimbs [16]. Consequently, a clear distinction between neck and trunk regions can no longer be made in snakes due to the complete absence of these landmark structures [17,16] and no traces of these have ever been uncovered in extant or fossil snakes. In fact, on pure anatomical grounds, it is unclear whether the ‘thoracic’ vertebrae in the snake could not be equally well classified as cervical. Indeed, in several snake species ventral hypapophyses, which often are considered a cervical characteristic, are present on very posterior vertebrae (suggestive of a cervical identity) and based on this it has been suggested that the snakes possess extremely long necks and possibly evolved through an extension of the neck region [59, 65, 17]. However, the presence of these hypapophyses is extremely variable among different snake clades, being anteriorly restricted in some taxa and almost universally present in others. A more stably positioned landmark, the *muscle cervicoquadratus*, a presumed pectoral girdle muscle homolog, has been suggested to mark the ancestral position of the pectoral girdle at the 11^th^ vertebra [66]. Therefore, the size of the snake’s cervical region and whether snakes evolved through transitional forms with short or elongated necks are controversial issues.

In vertebrates, *Hox* genes are expressed in a conserved pattern around the cervico-thoracic transition marking the position of the shoulder girdle [36,37]. Despite the fact that snakes seem to have partially lost the developmental responses to the *Hox* pattern, the expression patterns may still be informative with respect to the cervico-dorsal transition in modern snakes and the ancestral position of the shoulder girdle and forelimbs. The pattern of *Hoxc5, Hoxa6*, *Hoxb6*, *Hoxc6 *and *Hoxb8 *expression was compared between the bearded dragon lizard (*Pogona vitticeps*), which has a normal division between neck and trunk and the corn snake [4]. In the lizard the anterior boundaries of these genes all cluster within 4 vertebrae from the cervico-dorsal transition similar to observations in other species [36] the shoulder girdle position being marked by the expression of *Hoxc6*. In the corn snake the boundaries of these genes are similarly associated with the anterior part of the vertebral column between vertebrae 3 and 11 and cluster around the transition from the rib-less to the rib-bearing part of the vertebral column. This indicates that in modern snakes the cervico-dorsal transition is located in the anterior part of the vertebral column, as in lizards. Interestingly, the pattern is only slightly elongated in snakes; spanning 4 vertebrae between *Hoxb6* and *Hoxc6 *in the lizard and ~10 somites in the snake. This seems consistent with a higher speed of the somitogenesis clock and the production of more somites within this *Hox *expression zone but potentially also with a slight anterior shift of *Hox* expression during evolution, possibly to compensate in the transitional stages for an increased somitogenesis rate which also would affect the number of cervical vertebrae. The reflection of a typical cervico-dorsal transition in the *Hox *patterning, which displays the footprint of the elongation process, argues in favor of short-necked snake ancestors. Although this does not rule out an aquatic origin of snake, it still seems to contradict some of the standing scenarios and may strengthen the arguments for a terrestrial origin.

## CONCLUDING REMARKS

Here I provided an overview of the developmental mechanisms the evolution of which is likely to play a role in the evolution of a serpentine body plan. The main mechanisms identified to date function in elongation and regionalization during embryogenesis. It needs mentioning that so far the differences in these mechanisms are understood on a rather shallow level and many questions remain to be answered. For instance which mutations result in the increased pace of the somitogenesis oscillator? How are the *Hox13* genes regulated? And how has the interaction between the trunk *Hox *codes and their downstream targets evolved? The understanding of the evo-devo of the snake body plan requires a continued feeding on the field of developmental genetics, hopefully soon to be assisted by genome sequencing data. Such efforts will allow the large scale profiling of candidate regions for genomic signatures associated with the elusive mechanism of limb loss and, importantly, also widen the scope of research into non-candidate gene approaches, all together providing a further understanding of this striking example of tetrapod evolution.
